# Identification of Alfalfa Leaf Diseases Using Image Recognition Technology

**DOI:** 10.1371/journal.pone.0168274

**Published:** 2016-12-15

**Authors:** Feng Qin, Dongxia Liu, Bingda Sun, Liu Ruan, Zhanhong Ma, Haiguang Wang

**Affiliations:** 1 Department of Plant Pathology, China Agricultural University, Beijing, China; 2 College of Agriculture and Forestry Science and Technology, Hebei North University, Zhangjiakou, Hebei Province, China; 3 Institute of Microbiology, Chinese Academy of Sciences, Beijing, China; Universita del Salento, ITALY

## Abstract

Common leaf spot (caused by *Pseudopeziza medicaginis*), rust (caused by *Uromyces striatus*), Leptosphaerulina leaf spot (caused by *Leptosphaerulina briosiana*) and Cercospora leaf spot (caused by *Cercospora medicaginis*) are the four common types of alfalfa leaf diseases. Timely and accurate diagnoses of these diseases are critical for disease management, alfalfa quality control and the healthy development of the alfalfa industry. In this study, the identification and diagnosis of the four types of alfalfa leaf diseases were investigated using pattern recognition algorithms based on image-processing technology. A sub-image with one or multiple typical lesions was obtained by artificial cutting from each acquired digital disease image. Then the sub-images were segmented using twelve lesion segmentation methods integrated with clustering algorithms (including *K*_means clustering, fuzzy *C*-means clustering and *K*_median clustering) and supervised classification algorithms (including logistic regression analysis, Naive Bayes algorithm, classification and regression tree, and linear discriminant analysis). After a comprehensive comparison, the segmentation method integrating the *K*_median clustering algorithm and linear discriminant analysis was chosen to obtain lesion images. After the lesion segmentation using this method, a total of 129 texture, color and shape features were extracted from the lesion images. Based on the features selected using three methods (ReliefF, 1R and correlation-based feature selection), disease recognition models were built using three supervised learning methods, including the random forest, support vector machine (SVM) and *K*-nearest neighbor methods. A comparison of the recognition results of the models was conducted. The results showed that when the ReliefF method was used for feature selection, the SVM model built with the most important 45 features (selected from a total of 129 features) was the optimal model. For this SVM model, the recognition accuracies of the training set and the testing set were 97.64% and 94.74%, respectively. Semi-supervised models for disease recognition were built based on the 45 effective features that were used for building the optimal SVM model. For the optimal semi-supervised models built with three ratios of labeled to unlabeled samples in the training set, the recognition accuracies of the training set and the testing set were both approximately 80%. The results indicated that image recognition of the four alfalfa leaf diseases can be implemented with high accuracy. This study provides a feasible solution for lesion image segmentation and image recognition of alfalfa leaf disease.

## Introduction

Alfalfa (*Medicago sativa*) is an important forage grass containing various nutrients. The occurrence of disease in alfalfa plants has an important influence on the yield and quality of alfalfa hay, affecting the healthy development of the alfalfa industry [1]. There are more than ten types of alfalfa leaf diseases [2, 3]. Some of these diseases have similar symptoms, resulting in difficulties in achieving an accurate diagnosis and identifying the disease via naked-eye observations of symptoms or microscopic observations of causal agents. The diagnosis and identification of alfalfa diseases mainly rely on the experience of farmers, agricultural experts or agricultural technicians. The complexity of the disease symptoms and the limitations of personnel experience may lead to errors in judgment. The rapid, accurate identification and diagnosis of diseases will help to reduce yield losses and quality decline of alfalfa hay, resulting from the diseases. With the rapid development of computer technology and information technology, it is possible to utilize image-processing technology to diagnose and identify alfalfa leaf diseases quickly, accurately and automatically.

Image-processing technology has been applied to the recognition of many plant diseases [4–19]. The image-based recognition accuracy for plant diseases depends largely on the segmentation of the lesion images. Threshold-based image segmentation methods have been widely used in the segmentation of lesion images of diseased plants [20, 21]. However, there is usually great variance in color both between lesions of different diseases and between lesions from a disease at different stages. Therefore, it is very difficult to determine the appropriate threshold when threshold-based image segmentation methods are used to solving segmentation problems for plant disease images with complex colors. Image segmentation methods based on a fuzzy *C*-means clustering algorithm [22] or a *K_*means clustering algorithm [11, 15, 23] have been used to carry out lesion segmentation of plant disease images. Such segmentation methods must specify the number of clusters in advance. Inappropriate clustering number may lead to over-segmentation or under-segmentation of lesion images. However, a great computational cost is required to determine the appropriate number of clusters, especially for segmentation operations for high-pixel images. Supervised classification is a technique based on typical samples to deduce a functional equation for classification. Lesion segmentation of plant disease images can be effectively realized using the supervised classification method [24, 25]. However, the features of typical lesion regions and the features of typical health regions in a disease image cannot be obtained automatically and in a targeted fashion by only using a supervised classification method. Automatic segmentation of plant disease images can be effectively achieved by integrating a clustering algorithm and a supervised classification algorithm [26, 27].

There are color, texture and shape differences between lesion images from different plant diseases. Image recognition of plant diseases can be implemented using an appropriate pattern recognition algorithm based on color, texture and shape features of the lesion images [10, 11, 13, 17, 28]. Moreover, to reduce the complexity of the disease identification model and improve the model’s generalization ability, it is necessary to carry out feature selection according to the importance of features.

To the best of our knowledge, systematic studies on image recognition of alfalfa diseases have not yet been reported. In this study, automatic recognition of four common alfalfa leaf diseases including alfalfa common leaf spot (caused by *Pseudopeziza medicaginis*), alfalfa rust (caused by *Uromyces striatus*), alfalfa Leptosphaerulina leaf spot (caused by *Leptosphaerulina briosiana*) and alfalfa Cercospora leaf spot (caused by *Cercospora medicaginis*), was investigated based on acquired digital disease images. Of twelve segmentation methods integrating with clustering algorithms (including *K*_means clustering, fuzzy *C*-means clustering and *K*_median clustering) and supervised classification algorithms (including logistic regression analysis, Naive Bayes algorithm, classification and regression tree (CART) and linear discriminant analysis), the best image segmentation method was selected for further image processing and image recognition. After extraction of texture, color and shape features from the lesion images, feature selection was conducted using three different methods, i.e., the ReliefF method [29], the 1-rule (1R) method [30] and the correlation-based feature selection (CFS) method [31]. Based on the selected features, disease recognition models were built using three supervised learning methods including random forest, support vector machine (SVM) and *K*-nearest neighbor (KNN). Moreover, after the features used for building the optimal supervised model were transformed using principal component analysis (PCA), disease recognition semi-supervised models were built using a self-training algorithm based on Naive Bayes classifiers [32, 33]. After comparing the recognition results of each model, the optimal model was determined for disease image recognition. The aim of this study was to provide a solution for rapid and accurate identification of four alfalfa leaf diseases and provide some supports for the development of an automatic alfalfa leaf disease diagnosis system.

## Materials and Methods

### Image Acquisition

Infected alfalfa leaves with typical symptoms used in this study were sampled from the Langfang Forage Experimental Base, Institute of Animal Science, Chinese Academy of Agricultural Sciences and alfalfa fields in Xuanhua District, Zhangjiakou, Hebei Province, China. The study was conducted with the permission for the Langfang Forage Experimental Base given by Qinghua Yuan from Institute of Animal Science, Chinese Academy of Agricultural Sciences, Beijing, China. And the study was conducted with the permission for the alfalfa fields in Xuanhua District given by Dongxia Liu from College of Agriculture and Forestry Science and Technology, Hebei North University, Zhangjiakou, Hebei Province, China. All the diseased alfalfa leaves in the fields resulted from natural infections. The infected alfalfa leaves in the early stage of diseases were not sampled. Samples were taken to the laboratory and disease types of the leaves were determined mainly by using conventional diagnostic methods including naked-eye observations of disease symptoms and microscopic observation of morphological characteristics of causal agents. Images were captured with the lesion side of each diseased leaf facing up on a white background. When taking images, the leaves were expanded as flat as possible, and the camera lens was parallel with the plane of the leaves.

A total of 899 images with typical disease symptoms were acquired, including 76 images of alfalfa common leaf spot, 136 images of alfalfa rust, 231 images of alfalfa Leptosphaerulina leaf spot and 456 images of alfalfa Cercospora leaf spot. The image size was 4,256×2,832 pixels (jepg format). To reduce the workload of image analysis and focus on the regions of interest, a sub-image with one typical lesion or multiple typical lesions was obtained from each original disease image using artificial cutting. The size of a sub-image depended on the number of typical lesions and the size of each typical lesion. Using the sub-images, the image dataset of alfalfa common leaf spot comprising 76 sub-images, the image dataset of alfalfa rust comprising 136 sub-images, the image dataset of alfalfa Leptosphaerulina leaf spot comprising 231 sub-images, the image dataset of alfalfa Cercospora leaf spot comprising 456 sub-images and the aggregated image dataset comprising 899 sub-images, were constructed. These image datasets were used for segmentation of lesion images and evaluation of segmentation methods.

### Lesion Image Segmentation

**Fig 1:**
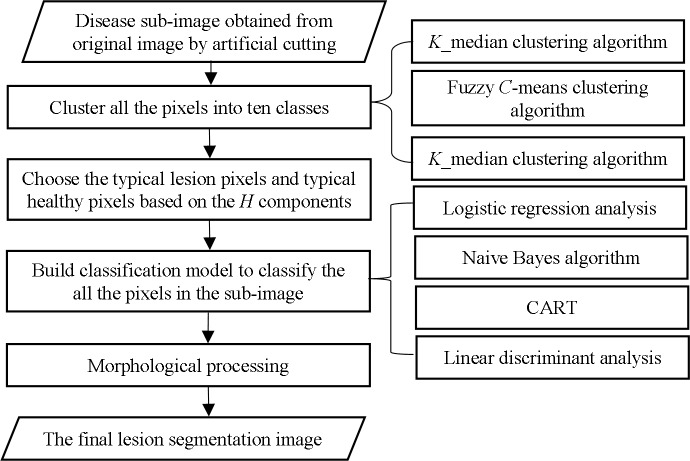
Work flow diagram of main steps for lesion image segmentation.

In this study, twelve lesion segmentation methods integrated with clustering algorithms (including *K*_means clustering, fuzzy *C*-means clustering and *K*_median clustering) and supervised classification algorithms (including logistic regression analysis, Naive Bayes algorithm, CART and linear discriminant analysis) were used to segment the sub-images, and then their segmentation effects were evaluated. The main steps for lesion image segmentation are shown in Fig 1.

Each obtained sub-image was converted from RGB color space into HSV color space and L*a*b* color space. In each pixel in the sub-image, the a* component value and the b* component value were regarded as the color features of the pixel. All pixels in the image were clustered into ten classes using *K*_median clustering, fuzzy *C*-means clustering and *K*_median clustering. The three clustering algorithms were carried out using the software MATLAB R2013b (MathWorks, Natick, MA, USA). For *K*_median clustering, the number of repetitions was set to three, and default values were used for the other parameters. For fuzzy *C*-means clustering, all the parameters with the default values were used. *K*_median clustering was implemented using Euclidean distance while the initial clustering seed was obtained using a random selection method. The maximum number of iterations was set to 1,000, the number of repetitions was set to three, and minimizing the sum of the intraclass distances was regarded as the clustering criterion.

After all pixels in a sub-image were clustered into ten classes using a clustering algorithm, the mean of the *H* components of all pixels in each class was calculated. Compared to healthy alfalfa leaves, the *H* components of the sub-images of the four alfalfa leaf diseases were smaller. Consequently, the pixels in the class with the minimum mean were treated as typical lesion pixels, and the pixels in the seven classes with the largest means were treated as typical healthy pixels. There is a transition region between the lesion region with typical symptoms and the typical healthy region, and *H* components are usually between the two regions. The pixels in the two remaining classes were treated as pixels that were not involved in building the pixel classification models. The typical lesion pixels and typical healthy pixels were labeled positive samples and negative samples, respectively, and these pixels constituted the training set for building pixel classification models. With a* component value and b* component value of each pixel in the training set as feature variables, pixel classification models to classify all the pixels in the sub-image were built using logistic regression analysis, Naive Bayes algorithm, CART and linear discriminant analysis, respectively. For each classification model, each pixel classified as lesion was assigned a value of 1 and each remaining pixel in the sub-image was assigned a value of 0. Thus, an initial binary lesion segmentation image was obtained. To avoid the influence of the white background, the pixels with *B* component values higher than 200 in the original sub-image were identified. Each pixel with *B* component value higher than 200 was assigned a value of 0, and each remaining pixel was assigned a value of 1 in the initial binary lesion segmentation image to achieve a binary image. A new binary image was obtained by multiplying this binary image with the initial binary lesion segmentation image. The hole filling operation was performed on this new binary image and the areas of all connected regions in the image were calculated. The connected regions with areas less than one-sixteenth of the maximum area were removed, and the final lesion segmentation image was obtained. If there were no pixels with *B* component values higher than 200, a hole filling operation was carried out on the initial binary lesion segmentation image. Subsequently, the areas of all connected regions in the image were calculated and the connected regions with the areas less than one-sixteenth of the maximum area were removed to give the final lesion segmentation image.

In the process of lesion segmentation, all pixels in a sub-image were classified as either lesion pixels or healthy pixels. Therefore, lesion segmentation is similar to binary classification problem in the field of pattern recognition, and the evaluation of segmentation effects can be carried out using methods for evaluating a binary classification model. Manual segmentation of a sub-image using the Adobe Photoshop CC software was conducted to determine the true class of each pixel. In comparison with manual segmentation method, Recall and Precision, two commonly used indices for evaluating classification models in the field of pattern recognition [34], were used to evaluate the twelve segmentation methods integrated with clustering algorithms and supervised classification algorithms. In this study, the two indices were calculated according to the following formulas: Recall = *N*_1_/*N*_2_ and Precision = *N*_1_/*N*_3_, where *N*_1_ was the total number of lesion pixels in a sub-image correctly classified by using a segmentation method integrated with a clustering algorithm and a supervised classification algorithm, *N*_2_ was the total number of lesion pixels in the sub-image classified using the manual segmentation method, and *N*_3_ was the total number of the pixels in the sub-image. Both Recall and Precision range from 0–1. Larger values of Recall and Precision indicate a better integrated segmentation method. The index “Score” combining Recall and Precision, is proposed in this study to evaluate the twelve segmentation methods and is calculated according to the following formula: Score = (Recall+Precision)/2. The Score also ranges from 0 to 1. Larger Score values demonstrate that the corresponding integrated segmentation method is better. Based on the image datasets described above, the three indices were used to evaluate the twelve integrated segmentation methods for achieving the best method to segment sub-images for further image recognition in this study.

After segmentation, in each final binary lesion segmentation image, each independent white region (i.e., connected component) was labeled a lesion, and the black background region was labeled the healthy region. The location of the smallest rectangle containing each lesion, namely, the independent white region, was determined. After multiplying each of color channels (*R*, *G* and *B*) of the original sub-image with the corresponding final binary lesion segmentation image, the obtained images were integrated into a new RGB image using the MATLAB system function “cat” to remove the background of the original sub-image and retain only the lesion regions. Based on the location information of each smallest rectangle containing a lesion, each rectangle was cut down from the new RGB image using the MATLAB system function “imcrop” to achieve multiple lesion images. For example, if there were two lesions in an original sub-image, two lesion images were achieved through the above operations. After segmentation using the best segmentation method based on the 899 sub-images of the four types of alfalfa leaf diseases, a total of 1,651 typical lesion images, each of which contained only one lesion, were obtained for further feature extraction, feature selection and disease image recognition. For building disease recognition models, the typical lesion images were divided into a training set and a testing set in a ratio of 2:1. The training set consisted of 1,100 lesion images including 111 lesion images of alfalfa common leaf spot, 267 lesion images of alfalfa rust, 371 lesion images of alfalfa Leptosphaerulina leaf spot and 351 lesion images of alfalfa Cercospora leaf spot. The testing set consisted of 551 lesion images including 56 lesion images of alfalfa common leaf spot, 133 lesion images of alfalfa rust, 185 lesion images of alfalfa Leptosphaerulina leaf spot and 177 lesion images of alfalfa Cercospora leaf spot.

### Feature Extraction and Normalization

A total of 129 texture, color and shape features were extracted from the 1,651 typical lesion images of the four alfalfa leaf diseases. The 90 extracted texture features included the seven Hu invariant moments (63 features), contrast (nine features), energy (nine features) and homogeneity (nine features) of the gray images of the nine components in RGB color space, HSV color space and L*a*b* color space. There were 30 color features including the first moments (nine features), the second moments (nine features) and the third moments (nine features) of the gray images of the nine components in RGB, HSV and L*a*b* color spaces, and three color ratios (*r*, *g* and *b*) of *R*, *G* and *B* components. Of the nine extracted shape features, circularity of disease lesion, complexity of disease lesion and the seven Hu invariant moments of the binary lesion image were included.

Hu invariant moments used to depict the texture features of an image are invariant to translation, rotation and scaling. Contrast is applied to measure the gray level of a pixel in comparison with the neighbor pixels in an image, energy is a measure of the consistency of an image, and homogeneity is used to measure the spatial closeness of elements with the diagonal distribution in a co-occurrence matrix [35]. Circularity denotes the degree that a lesion region is circular, and a bigger value indicates that the lesion region is more circular [11]. Complexity refers to the complexity and discrete degree of a lesion region, and a bigger value indicates the lesion region with higher complexity and greater discrete degree [11]. The seven Hu invariant moments were calculated using the calculation formulas as described in [36]. The other extracted features were calculated according to the formulas shown in Table 1.

**Table 1:** Extracted image features (excluding Hu invariant moments) and calculation formulas.

Feature parameter	Calculation formula	Reference
Contrast	$\sum_{i=1}^{M}\sum_{j=1}^{M}{\left(i-j\right)}^{2}p_{ij}$, where *M*×*M* denotes the size of a co-occurrence matrix, *M* = 1, 2, …, and *p*_*ij*_ denotes the quotient of the element (*i*, *j*) of a co-occurrence matrix divided by the sum of the elements of the co-occurrence matrix.	[35]
Energy	$\sum_{i=1}^{M}\sum_{j=1}^{M}p_{ij}^{2}$, where *M*×*M* denotes the size of a co-occurrence matrix, *M* = 1, 2, …, and *p*_*ij*_ denotes the quotient of the element (*i*, *j*) of a co-occurrence matrix divided by the sum of the elements of the co-occurrence matrix.	[35]
Homogeneity	$\sum_{i=1}^{M}\sum_{j=1}^{M}\frac{p_{ij}}{1+\left|i-j\right|}$, where *M*×*M* denotes the size of a co-occurrence matrix, *M* = 1, 2, …, and *p*_*ij*_ denotes the quotient of the element (*i*, *j*) of a co-occurrence matrix divided by the sum of the elements of the co-occurrence matrix.	[35]
First moment	$\frac{1}{L}\sum_{i=0}^{L-1}f_{i}p\left(f_{i}\right)$, where *μ*_1_, *μ*_2_ and *μ*_3_ refer to the first moment, second moment and third moment, respectively, *f*_*i*_ represents a random variable of gray level, *p*(*f*_*i*_) represents the gray level histogram of an image region, *i* = 0, 1, 2, …, *L*-1, and *L* is the number of different gray levels.	[37]
Second moment	${\left[\frac{1}{L}\sum_{i=0}^{L-1}{\left(f_{i}-\mu_{1}\right)}^{2}p\left(f_{i}\right)\right]}^{\frac{1}{2}}$, where *μ*_1_, *μ*_2_ and *μ*_3_ refer to the first moment, second moment and third moment, respectively, *f*_*i*_ represents a random variable of gray level, *p*(*f*_*i*_) represents the gray level histogram of an image region, *i* = 0, 1, 2, …, *L*-1, and *L* is the number of different gray levels.	[37]
Third moment	${\left[\frac{1}{L}\sum_{i=0}^{L-1}{\left(f_{i}-\mu_{1}\right)}^{3}p\left(f_{i}\right)\right]}^{\frac{1}{3}}$, where *μ*_1_, *μ*_2_ and *μ*_3_ refer to the first moment, second moment and third moment, respectively, *f*_*i*_ represents a random variable of gray level, *p*(*f*_*i*_) represents the gray level histogram of an image region, *i* = 0, 1, 2, …, *L*-1, and *L* is the number of different gray levels.	[37]
Color ratio *r*	$\frac{R}{R+G+B}$	[11]
Color ratio *g*	$\frac{G}{R+G+B}$	[11]
Color ratio *b*	$\frac{B}{R+G+B}$	[11]
Circularity	$\frac{4\pi S}{L^{2}}$, where *S* and *L* represent the area and perimeter of a lesion region, respectively.	[11]
Complexity	$\frac{L^{2}}{S}$, where *S* and *L* represent the area and perimeter of a lesion region, respectively.	[11]

Because of the great differences between the ranges of extracted features, which may impact the accuracies of disease recognition models, the values of each extracted feature were normalized to the range of 0–1 using the following formula: $X_{\text{norm}}^{i}=\left(X^{i}-X_{\min}^{i}\right)/\left(X_{\max}^{i}-X_{\min}^{i}\right)$, where $X_{\text{norm}}^{i}$ was the value of the *i*th feature after normalization and *X*^*i*^, $X_{\min}^{i}$ and $X_{\max}^{i}$ were the value of the *i*th feature, the minimum value and the maximum value of the feature before normalization, respectively.

### Feature Selection

To reduce the complexity of image recognition resulting from excessive features and improve the accuracy and applicability of image recognition methods, the extracted features were screened after feature normalization. Based on the training set including 1,100 lesion images described above, feature selection was conducted using the ReliefF method, the 1R method and the CFS method.

For the ReliefF method, a high weight was assigned to a feature that has a high correlation with categories, and a feature with a higher weight indicates that this feature is more important. For the 1R method, the classification accuracy is calculated with each feature as the input of the 1R classifier successively and is used to evaluate the importance of the feature. Higher classification accuracy indicates that the corresponding feature is more important. The CFS method is unlike the ReliefF method and the 1R method, and is aimed to obtain the optimal feature subset. The correlation between the optimal feature subset and dependent variable should be as high as possible. Meanwhile, the correlations among the features in the optimal feature subset should be as small as possible. In this study, the three methods for feature selection, including the ReliefF method, the 1R method and the CFS method, were all implemented using the open source software Weka (Waikato Environment for Knowledge Analysis) 3.7, developed by The University of Waikato in Hamilton, New Zealand. The default values were used for all the parameters involved in the methods. The importance ranking of each feature for classification and recognition could be obtained using the ReliefF method and the 1R method, respectively. A higher ranking for a feature indicates that it is more likely to yield better recognition results if used to build the recognition model. To find the best combination of features, according to the importance ranking of each feature for classification and recognition, the top *N* (*N* = 1, 2, 3, …, 129) features were treated as inputs for the disease recognition models based on random forest, SVM and KNN. According to the recognition accuracies of the training set and the testing set, the best top *N* features were selected as the best feature combination to build the disease recognition models. For the CFS method, the best feature combination, namely, the optimal feature subset, was obtained directly for modeling.

### Building of Disease Recognition Models

After the segmentation, feature extraction, feature normalization and feature selection described above, disease recognition models were built based on the 1,651 typical lesion images of the four alfalfa leaf diseases using three supervised learning methods including random forest, SVM and KNN. All models were built using the MATLAB R2013b software. The recognition accuracies of both the training set and the testing set were calculated and used to evaluate the disease recognition models.

Random forest is a combination model composed of a number of fully grown decision trees [38]. Each decision tree produces a predictive value, and the final prediction result of the model can be determined by voting. To a certain extent, the classification effects of a random forest depend on the number of decision trees that constitute the model. Consequently, it is necessary to determine the optimal number of decision trees by testing a variety of values based on the classification results of the random forests. To build a disease recognition model based on the random forest method, the number of decision trees was assigned as 10, 20, 30, 40, 50, 60, 70, 80, 90, and 100, and the optimal number of decision trees was determined according to the recognition results of the model. The number of features randomly selected by each decision tree was set as the arithmetic square root of the total number of features. If the arithmetic square root was a decimal, the value obtained by rounding up the decimal was treated as the number of features randomly selected by each decision tree.

SVM can be well applied to high-dimensional data [39, 40]. It has been widely used in image recognition of plant disease [11, 20, 28, 41]. In this study, SVM models for disease image recognition were built with a radial basis function as the kernel function using C-SVM in the LIBSVM package developed by Chih-Jen Lin Group from Taiwan, China [42]. For each SVM model, both the optimal penalty parameter *C* and the optimal kernel function parameter *g* were searched using the grid search algorithm in the range of 2^−10^–2^10^ with a searching step of 0.4. The recognition accuracies were calculated at all points within the grid by running three complete cross validations based on the training set. The values of *C* and *g* were selected as the optimal parameters as the recognition accuracy was the highest, and were recorded as *C*_best_ and *g*_best_, respectively.

The KNN algorithm treats each sample as a point in a multidimensional space, and a point in the testing set is assigned to a class that most of the *K* points nearest to that point in the training set belong to [43, 44]. The distance of that point to each of the *K* points is commonly measured by Euclidean distance. An appropriate value of *K* is the key to high classification accuracy using the KNN algorithm. In this study, to build the KNN models for image recognition using Euclidean distance, the *K* values were set as 5, 9 and 13, respectively, and the optimal value of *K* was determined according to the recognition results of the models.

For the supervised learning methods, the true class that each sample in the training set belongs to is known. In other words, all samples in the training set are labeled samples. In some cases, the cost of obtaining training samples is low, but the cost of determining the true class of the training samples is very high, which requires a large amount of manpower and material resources. When a small number of samples in the training set are labeled, a recognition model can be built using a semi-supervised learning method. In practice, when many disease images are acquired with lower costs, the experts in the corresponding field just need to make artificial recognition and classification of a small number of disease images. Disease recognition models can be built using semi-supervised learning methods, which will greatly reduce the costs of building a plant disease automatic recognition system. In this study, the features used to build the optimal supervised model were transformed using PCA and the disease recognition semi-supervised models were built using a self-training algorithm based on Naive Bayes classifiers [32, 33]. In this method, an initial classifier is built based on the given labeled samples and used to predict the unlabeled samples in the training set. The prediction labels with high confidence in the classifier and their corresponding samples are added to a dataset comprising the labeled samples from the training set. Subsequently, based on the new dataset comprising the labeled samples, a new classifier is built. The above process continues until a certain criterion is reached. The criterion may be the number of iterations reaching the maximum number of iterations or the number of labeled samples reaching the set ratio, etc. In this study, based on the same training set and testing set as used for building the supervised models described above, disease recognition semi-supervised models were built with ratios of the labeled and unlabeled samples in the training set equal to 2:1, 1:1 and 1:2. The first *n* principal components were successively used to build the disease recognition semi-supervised models, and the corresponding recognition accuracies of the training set and the testing set were obtained. According to the accuracies, the recognition effects of the models were evaluated, and the optimal number of principal components was determined. The above disease recognition semi-supervised models were built using the R3.1.2 software and the function “SelfTrain” in the package “DMwR” as the default values were used for the model parameters.

## Results

### Image Segmentation Results

Based on the image datasets described above, the comparison results of the twelve segmentation methods integrated with the clustering algorithms and the supervised classification algorithms are shown in Table 2.

**Table 2:** Performance evaluations of the twelve segmentation methods based on the sub-images of four alfalfa leaf diseases.

Image dataset	Clustering method	Supervised classification method	Recall		Precision		Score	
			Mean	Median	Mean	Median	Mean	Median
Image dataset of alfalfa common leaf spot	*K*_means clustering algorithm	Logistic regression analysis	0.6443	0.6727	0.8940	0.9127	0.7691	0.7925
		Naive Bayes algorithm	0.6318	0.6379	0.8922	0.9047	0.7620	0.7644
		CART	0.5547	0.5683	0.8716	0.8829	0.7132	0.7300
		Linear discriminant analysis	0.7694	0.7981	0.9235	0.9392	0.8465	0.8743
	Fuzzy *C*-means clustering algorithm	Logistic regression analysis	0.6117	0.6455	0.8859	0.9119	0.7488	0.7783
		Naive Bayes algorithm	0.5725	0.5894	0.8785	0.8876	0.7255	0.7484
		CART	0.4856	0.4684	0.8570	0.8686	0.6713	0.6639
		Linear discriminant analysis	0.7389	0.8036	0.9155	0.9357	0.8272	0.8736
	*K*_median clustering algorithm	Logistic regression analysis	0.6989	0.7261	0.9010	0.9272	0.7999	0.8291
		Naive Bayes algorithm	0.6850	0.6785	0.8982	0.9234	0.7916	0.7949
		CART	0.6153	0.5926	0.8806	0.9001	0.7479	0.7435
		Linear discriminant analysis	0.7905	0.8199	0.9220	0.9366	0.8562	0.8810
Image dataset of alfalfa rust	*K*_means clustering algorithm	Logistic regression analysis	0.7508	0.7714	0.9459	0.9568	0.8484	0.8626
		Naive Bayes algorithm	0.7200	0.7354	0.9396	0.9517	0.8298	0.8444
		CART	0.7021	0.7370	0.9372	0.9518	0.8197	0.8413
		Linear discriminant analysis	0.8303	0.8376	0.9583	0.9639	0.8943	0.9013
	Fuzzy *C*-means clustering algorithm	Logistic regression analysis	0.6741	0.6772	0.9338	0.9423	0.8039	0.8091
		Naive Bayes algorithm	0.6366	0.6424	0.9266	0.9386	0.7816	0.7872
		CART	0.5998	0.6156	0.9197	0.9314	0.7598	0.7721
		Linear discriminant analysis	0.8051	0.8116	0.9549	0.9609	0.8800	0.8870
	*K*_median clustering algorithm	Logistic regression analysis	0.8166	0.8384	0.9542	0.9644	0.8854	0.9025
		Naive Bayes algorithm	0.8019	0.8288	0.9458	0.9595	0.8738	0.8968
		CART	0.7915	0.8341	0.9475	0.9640	0.8695	0.9019
		Linear discriminant analysis	0.8516	0.8596	0.9606	0.9671	0.9061	0.9137
Image dataset of alfalfa Leptosphaerulina leaf spot	*K*_means clustering algorithm	Logistic regression analysis	0.8329	0.8736	0.9634	0.9722	0.8982	0.9248
		Naive Bayes algorithm	0.8561	0.8908	0.9635	0.9713	0.9098	0.9335
		CART	0.7665	0.7933	0.9571	0.9697	0.8618	0.8803
		Linear discriminant analysis	0.9002	0.9285	0.9657	0.9716	0.9330	0.9510
	Fuzzy *C*-means clustering algorithm	Logistic regression analysis	0.8102	0.8334	0.9622	0.9733	0.8862	0.9040
		Naive Bayes algorithm	0.8181	0.8407	0.9623	0.9718	0.8902	0.9078
		CART	0.7188	0.7458	0.9536	0.9674	0.8362	0.8569
		Linear discriminant analysis	0.8900	0.9170	0.9652	0.9723	0.9276	0.9441
	*K*_median clustering algorithm	Logistic regression analysis	0.8919	0.9255	0.9613	0.9691	0.9266	0.9481
		Naive Bayes algorithm	0.9091	0.9480	0.9583	0.9626	0.9337	0.9565
		CART	0.8629	0.9156	0.9556	0.9656	0.9093	0.9409
		Linear discriminant analysis	0.9287	0.9495	0.9636	0.9690	0.9462	0.9583
Image dataset of alfalfa Cercospora leaf spot	*K*_means clustering algorithm	Logistic regression analysis	0.5851	0.6044	0.8250	0.8471	0.7051	0.7172
		Naive Bayes algorithm	0.5296	0.5394	0.8044	0.8173	0.6670	0.6823
		CART	0.4240	0.4184	0.7627	0.7672	0.5933	0.5908
		Linear discriminant analysis	0.7656	0.7824	0.8932	0.9041	0.8294	0.8431
	Fuzzy *C*-means clustering algorithm	Logistic regression analysis	0.5808	0.5954	0.8231	0.8401	0.7019	0.7184
		Naive Bayes algorithm	0.5089	0.5184	0.7968	0.8122	0.6529	0.6680
		CART	0.4184	0.4120	0.7620	0.7667	0.5902	0.5878
		Linear discriminant analysis	0.7508	0.7695	0.8877	0.9027	0.8192	0.8330
	*K*_median clustering algorithm	Logistic regression analysis	0.6237	0.6330	0.8362	0.8612	0.7300	0.7488
		Naive Bayes algorithm	0.5705	0.5885	0.8171	0.8373	0.6938	0.7149
		CART	0.4876	0.4702	0.7824	0.7985	0.6350	0.6292
		Linear discriminant analysis	0.7786	0.7938	0.8951	0.9109	0.8369	0.8496
Aggregated image dataset	*K*_means clustering algorithm	Logistic regression analysis	0.6789	0.7040	0.8846	0.9116	0.7818	0.8057
		Naive Bayes algorithm	0.6510	0.6474	0.8730	0.8960	0.7620	0.7690
		CART	0.5650	0.5728	0.8481	0.8791	0.7065	0.7138
		Linear discriminant analysis	0.8105	0.8317	0.9242	0.9419	0.8674	0.8870
	Fuzzy *C*-means clustering algorithm	Logistic regression analysis	0.6567	0.6633	0.8808	0.9044	0.7687	0.7814
		Naive Bayes algorithm	0.6132	0.6041	0.8658	0.8830	0.7395	0.7412
		CART	0.5288	0.5180	0.8430	0.8679	0.6859	0.6922
		Linear discriminant analysis	0.7940	0.8184	0.9201	0.9388	0.8571	0.8777
	*K*_median clustering algorithm	Logistic regression analysis	0.7282	0.7648	0.8916	0.9270	0.8099	0.8468
		Naive Bayes algorithm	0.7022	0.7042	0.8795	0.9119	0.7908	0.8028
		CART	0.6407	0.6714	0.8600	0.9035	0.7504	0.7868
		Linear discriminant analysis	0.8294	0.8514	0.9249	0.9424	0.8771	0.8997

Note: The aggregated image dataset was obtained after aggregation of four image datasets of alfalfa common leaf spot, alfalfa rust, alfalfa Leptosphaerulina leaf spot and alfalfa Cercospora leaf spot.

For the image dataset of alfalfa common leaf spot, when the segmentation method integrated with *K*_median clustering algorithm and linear discriminant analysis was used, the highest Scores with a mean of 0.8562 and the median of 0.8810 were obtained, and the highest Recalls with a mean of 0.7905 and the median of 0.8199 were also obtained. When the segmentation method integrating with *K*_means clustering algorithm and linear discriminant analysis was used based on the image dataset, the highest Precisions with a mean of 0.9235 and the median of 0.9392 were obtained.

For the image dataset of alfalfa rust, when the segmentation method integrated with *K*_median clustering algorithm and linear discriminant analysis was used, the highest values of Scores, Recalls and Precisions were obtained. The results showed that the mean of Scores was 0.9061 and the median of Scores was 0.9137, that the mean of Recalls was 0.8516 and the median of Recalls was 0.8596 and that the mean of Precisions was 0.9606 and the median of Precisions was 0.9671.

For the image dataset of alfalfa Leptosphaerulina leaf spot, when the segmentation method integrated with *K*_median clustering algorithm and linear discriminant analysis was used, the highest values of Scores and Recalls were obtained. The results showed that the mean of Scores was 0.9462 and the median of Scores was 0.9583 and that the mean of Recalls was 0.9287 and the mean of Recalls was 0.9495. For this image dataset, when the segmentation method integrated with *K*_means clustering algorithm and linear discriminant analysis was used, the highest mean of Precisions was obtained and its value was 0.9657. For this image dataset, when the segmentation method integrating with fuzzy *C*-means clustering algorithm and logistic regression analysis was used, the highest median of Precisions was obtained, and its value was 0.9733.

For the image dataset of alfalfa Cercospora leaf spot, when the segmentation method integrated with *K*_median clustering algorithm and linear discriminant analysis was used, the highest values of Scores, Recalls and Precisions were obtained. The mean and the median of Scores were 0.8369 and 0.8496, respectively. The mean and the median of Recalls were 0.7786 and 0.7938, respectively, and the mean and the median of Precisions were 0.8951and 0.9109, respectively.

For the aggregated image dataset comprising 899 sub-images of the four alfalfa leaf diseases, when the segmentation method integrated with *K*_median clustering algorithm and linear discriminant analysis was used, the highest values of Scores, Recalls and Precisions were obtained. The results showed that the mean and the median of Scores were 0.8771 and 0.8997, respectively, that the mean and the median of Recalls were 0.8294 and 0.8514, respectively and that the mean and the median of Precisions were 0.9249 and 0.9424, respectively.

**Fig 2:**
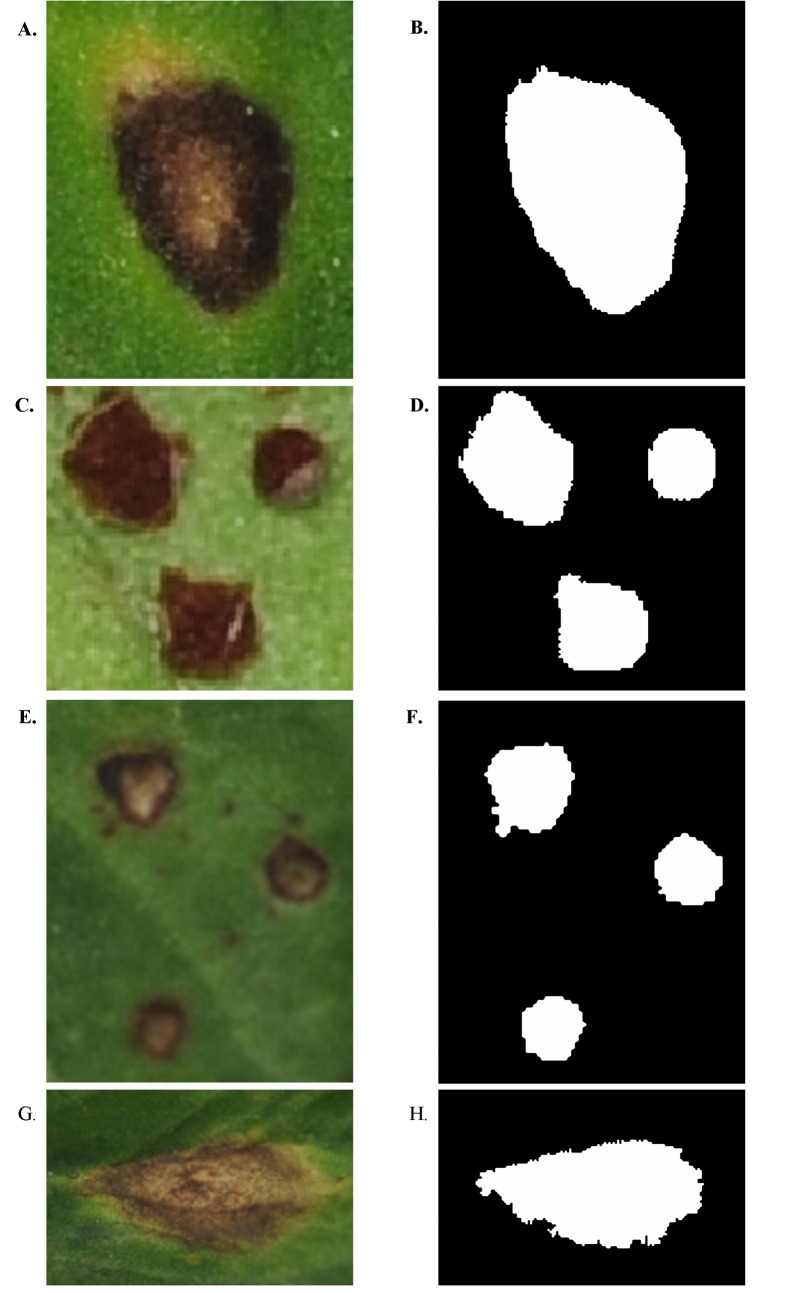
Results of automatic segmentation of sub-images of four alfalfa leaf diseases using the segmentation method integrated with *K*_ median clustering algorithm and linear discriminant analysis. A: Sub-image of alfalfa common leaf spot. B: Image after segmentation of alfalfa common leaf spot. C: Sub-image of alfalfa rust. D: Image after segmentation of alfalfa rust. E: Sub-image of alfalfa Leptosphaerulina leaf spot. F: Image after segmentation of alfalfa Leptosphaerulina leaf spot. G: Sub-image of alfalfa Cercospora leaf spot. H: Image after segmentation of alfalfa Cercospora leaf spot.

In summary, when the segmentation method integrated with *K*_median clustering algorithm and linear discriminant analysis was used, the segmentation effects for the sub-images of the four alfalfa leaf diseases were best. The segmentation results of the sub-images of the four alfalfa leaf diseases using the segmentation method integrated with *K*_ median clustering algorithm and linear discriminant analysis are shown in Fig 2. Using this segmentation method, all lesions in the original sub-images were effectively segmented. The results indicated that this segmentation method could effectively implement the automatic segmentation of sub-images of the four alfalfa leaf diseases. Therefore, lesion segmentation was implemented using the segmentation method integrated with *K*_median clustering algorithm and linear discriminant analysis for further feature extraction, feature normalization, feature selection and building of disease recognition models in this study.

### Feature Selection Results Using the Methods of ReliefF, 1R and CFS

For convenience, each extracted feature was given a name, and the names of the 129 image features extracted are listed in Table 3. φLab_L1 denoted the first Hu invariant moment of the gray image of the L* component in L*a*b* color space, φshape1 denoted the first Hu invariant moment of the binary lesion image, the first moment RGB_R denoted the first moment of the gray image of the *R* component in RGB color space, Color ratio RGB_R denoted the color ratio *r* of the *R* component in RGB color space, Contrast RGB_R denoted the contrast of the gray image of the *R* component in RGB color space, Energy RGB_R denoted the energy of the gray image of the *R* component in RGB color space and Homogeneity RGB_R denoted the homogeneity of the gray image of the *R* component in RGB color space. The remaining feature names can be deduced by analogy.

**Table 3:** Names of image features extracted and results of feature selection using the ReliefF method, the 1R method and the CFS method.

Feature name	Feature ranking based on the ReliefF method	Feature ranking based on the 1R method	Feature name	Feature ranking based on the ReliefF method	Feature ranking based on the 1R method	Feature name	Feature ranking based on the ReliefF method	Feature ranking based on the 1R method
φLab_L1	61	81	φHSV_H2	71	30	Second moment RGB_B	4	127
φLab_L2	77	103	φHSV_H3	101	40	Second moment HSV_H*	32	55
φLab_L3	102	106	φHSV_H4	85	43	Second moment HSV_S	38	67
φLab_L4	87	128	φHSV_H5	106	24	Second moment HSV_V	50	114
φLab_L5	95	113	φHSV_H6	88	28	Second moment Lab_L	28	18
φLab_L6	94	112	φHSV_H7	129	49	Second moment Lab_b	14	9
φLab_L7	126	126	φHSV_S1*	75	38	Second moment Lab_a	41	83
φLab_a1*	45	1	φHSV_S2	100	41	Third moment RGB_R	49	48
φLab_a2	70	31	φHSV_S3	110	72	Third moment RGB_G	54	53
φLab_a3	84	77	φHSV_S4	111	75	Third moment RGB_B	42	118
φLab_a4	79	58	φHSV_S5	113	65	Third moment HSV_H	44	76
φLab_a5	115	123	φHSV_S6	112	50	Third moment HSV_S*	36	69
φLab_a6	120	98	φHSV_S7	118	88	Third momentHSV_V	53	110
φLab_a7	116	124	φHSV_V1	63	74	The third moment Lab_L	30	19
φLab_b1	56	64	φHSV_V2	74	85	Third moment Lab_b	13	11
φLab_b2	68	46	φHSV_V3	96	102	Third moment Lab_a	39	79
φLab_b3	105	42	φHSV_V4	83	109	Contrast RGB_R	59	66
φLab_b4	119	61	φHSV_V5	98	104	Energy RGB_R	35	71
φLab_b5	117	121	φHSV_V6	90	86	Homogeneity RGB_R	18	25
φLab_b6	128	63	φHSV_V7	124	111	Contrast RGB_G	64	100
φLab_b7	114	122	Circularity*	2	5	Energy RGB_G	55	73
φRGB_R1	62	68	Complexity*	69	4	Homogeneity RGB_G	22	39
φRGB_R2	73	80	φshape1*	66	37	Contrast RGB_B	21	52
φRGB_R3	97	99	φshape2	72	44	Energy RGB_B*	1	34
φRGB_R4	82	91	φshape3	80	59	Homogeneity RGB_B	16	47
φRGB_R5	99	107	φshape4	81	32	Contrast HSV_H	47	6
φRGB_R6	89	94	φshape5	107	97	Energy HSV_H	6	27
φRGB_R7	125	101	φshape6	93	45	Homogeneity HSV_H	31	3
φRGB_G1	51	90	φshape7	127	108	Contrast HSV_S	33	16
φRGB_G2	76	62	First moment RGB_R	25	17	Energy HSV_S*	52	54
φRGB_G3	104	119	First moment RGB_G*	27	29	Homogeneity HSV_S*	9	8
φRGB_G4	86	120	First moment RGB_B*	24	36	Contrast HSV_V	60	70
φRGB_G5	92	93	Color ratio RGB_R*	3	14	Energy HSV_V	37	84
φRGB_G6	91	95	Color ratio RGB_G*	10	12	Homogeneity HSV_V	19	23
φRGB_G7	123	125	Color ratio RGB_B	23	92	Contrast Lab_L	67	87
φRGB_B1	48	78	First moment HSV_H*	8	33	Energy Lab_L	46	96
φRGB_B2	78	60	First moment HSV_S	7	51	Homogeneity Lab_L*	20	22
φRGB_B3	109	116	First moment HSV_V*	26	21	Contrast Lab_a*	58	2
φRGB_B4	103	117	First moment Lab_L	29	20	Energy Lab_a	5	26
φRGB_B5	121	105	First moment Lab_b*	15	10	Homogeneity Lab_a	43	15
φRGB_B6	108	89	First moment Lab_a	40	82	Contrast Lab_b	65	7
φRGB_B7	122	129	Second moment RGB_R	34	115	Energy Lab_b	11	35
φHSV_H1*	12	13	Second moment RGB_G*	17	57	Homogeneity Lab_b	57	56

Note:

* Features marked with an asterisk in the table were selected based on the CFS method.

The results of feature selection using the ReliefF method, 1R method and CFS method are shown in Table 3. The selection results of both the ReliefF method and the 1R method were the importance ranking of each feature for disease recognition. As shown in Table 3, there were great differences between the importance rankings of features obtained using the ReliefF method and the 1R method. The top 10 features with the highest recognition importance selected using the ReliefF method successively were Energy RGB_B, Circularity, Color ratio RGB_R, second moment RGB_B, Energy Lab_a, Energy HSV_H, first moment HSV_S, first moment HSV_H, Homogeneity HSV_S and Color ratio RGB_G, which included four texture features, one shape feature and five color features. The top 10 features with the highest recognition importance selected using the 1R method in sequence were φLab_a1, Contrast Lab_a, Homogeneity HSV_H, Complexity, Circularity, Contrast HSV_H, Contrast Lab_b, Homogeneity HSV_S, second moment Lab_b and first moment Lab_b, which included six texture features, two shape features and two color features. Only two features, Circularity and Homogeneity HSV_S, were simultaneously selected in the top 10 features with the highest recognition importance using the ReliefF method and the 1R method. The best feature combination (i.e., the optimal feature subset) obtained using the CFS method consisted of 21 features including φLab_a1, φHSV_H1, φHSV_S1, Circularity, Complexity, φshape1, first moment RGB_G, first moment RGB_B, Color ratio RGB_R, Color ratio RGB_G, first moment HSV_H, first moment HSV_V, first moment Lab_b, second moment RGB_G, second moment HSV_H, third moment HSV_S, Energy RGB_B, Energy HSV_S, Homogeneity HSV_S, Homogeneity Lab_L and Contrast Lab_a.

### Built Disease Recognition Models and Comparison of Recognition Results

#### Recognition Results of Disease Recognition Models Based on Random Forest

The recognition results of the random forest models based on the selected features using the ReliefF method, the 1R method or the CFS method are shown in Table 4. The results showed that when the ReliefF method was used to select the features, with the increase of the number of decision trees, the recognition accuracies of the training set and the testing set for the built random forest models fluctuated by 0%-2.18%, and the number of applied features changed in a range of 52–74. The optimal random forest model was built with the number of decision trees equal to 70 based on the top 62 features in the importance ranking for recognition, and this model was recorded as Model 1. For Model 1, the recognition accuracy of the training set was 100% and the recognition accuracy of the testing set was 92.74%. When the 1R method was used for feature selection, with an increase in the number of decision trees, the recognition accuracies of the training set and the testing set for the built random forest models fluctuated by 0%-2.00%, and the number of applied features changed in a range of 76–129. The optimal random forest model was built with the number of decision trees equal to 60 based on the top 128 features in the importance ranking and was recorded as Model 2. For Model 2, the recognition accuracy of the training set was 100% and the recognition accuracy of the testing set was 91.29%. When the CFS method was applied to feature selection, with the increase of the number of decision trees, the recognition accuracies of the training set and the testing set for the built random forest models fluctuated by 0%-2.18%. The optimal random forest model was built with the number of decision trees equal to 60 based on the 21 selected features, and this model was recorded as Model 3. For Model 3, the recognition accuracy of the training set was 100% and the recognition accuracy of the testing set was 90.20%. As shown in Table 4, with increasing number of decision trees, the recognition accuracies of the training set and the testing set for the built random forest models fluctuated within a small range, indicating that the number of decision trees had little influence on the recognition results of the random forest models in this study. Considering the recognition accuracies of the training set and the testing set and the number of applied features for modeling, the optimality ranking of the three optimal models was Model 1, Model 3, and Model 2.

**Table 4:** Recognition results for four alfalfa leaf diseases using random forest models based on selected features using the ReliefF method, the 1R method and the CFS method.

Number of decision trees	ReliefF method			1R method			CFS method		
	Recognition accuracy of training set (%)	Recognition accuracy of testing set (%)	Number of applied features	Recognition accuracy of training set (%)	Recognition accuracy of testing set (%)	Number of applied features	Recognition accuracy of training set (%)	Recognition accuracy of testing set (%)	Number of applied features
10	99.82	90.56	74	99.73	89.29	90	99.64	89.29	21
20	99.91	91.47	57	99.91	90.20	88	99.91	88.57	21
30	100	92.38	52	99.91	90.56	129	99.91	88.75	21
40	100	92.56	61	100	90.56	126	100	89.47	21
50	100	92.38	59	99.91	90.74	76	100	88.02	21
60	100	92.20	65	100	91.29	128	100	90.20	21
70	100	92.74	62	100	90.74	119	100	89.11	21
80	100	92.56	57	100	90.56	105	100	88.38	21
90	100	92.38	55	100	90.93	107	100	88.57	21
100	100	92.20	54	100	90.93	114	100	89.11	21

Note: For each number of decision trees, only the best random forest model for the recognition of the four alfalfa leaf diseases is shown when the features were selected using the ReliefF method or 1R method.

#### Recognition Results of Disease Recognition Models Based on SVM

The recognition results of the SVM models based on the selected features busing the ReliefF method, the 1R method and the CFS method are shown in Table 5. The results showed that when the ReliefF method was used to select the features, the optimal SVM model was built based on the top 45 features in the importance ranking for recognition, and this model was recorded as Model 4 with the optimal parameters *C*_best_ and *g*_best_ of 6.964 and 0.435. For Model 4, the recognition accuracy of the training set was 97.64% and the recognition accuracy of the testing set was 94.74%. When the 1R method was used to conduct feature selection, the optimal SVM model was built based on the top 122 features in the importance ranking for recognition, and this model was recorded as Model 5, with *C*_best_ equal to 36.758 and *g*_best_ equal to 0.144. For Model 5, the recognition accuracy of the training set was 97.91% and the recognition accuracy of the testing set was 94.37%. When the CFS method was used for feature selection, the SVM model built based on the 21 selected features was recorded as Model 6, with *C*_best_ equal to 21.112 and *g*_best_ equal to 0.758. For Model 6, the recognition accuracy of the training set was 95.18% and the recognition accuracy of the testing set was 91.83%. Considering the recognition accuracies of the training set and the testing set and the number of applied features for modeling, the optimality ranking of the three models shown in Table 5 was Model 4, Model 6, and Model 5.

**Table 5:** Recognition results for four alfalfa leaf diseases using SVM models based on selected features using the ReliefF method, the 1R method and the CFS method.

Model	Feature selection method	*C*_best_	*g*_best_	Recognition accuracy of training set (%)	Recognition accuracy of testing set (%)	Number of applied features
Model 4	The ReliefF method	6.964	0.435	97.64	94.74	45
Model 5	The 1R method	36.758	0.144	97.91	94.37	122
Model 6	The CFS method	21.112	0.758	95.18	91.83	21

Note: Only the best SVM model for the image recognition of the four alfalfa leaf diseases is shown when the features were selected using the ReliefF method or 1R method.

#### Recognition Results of Disease Recognition Models Based on KNN

The recognition results of the KNN models based on the selected features using the ReliefF method, the 1R method and the CFS method are shown in Table 6. The results showed that when the ReliefF method was used to select features, with the increase in the value of *K*, the recognition accuracies of the training set and the testing set for the built KNN models also fluctuated by 0%-3.55%. The optimal KNN model was built with a *K* value of 5 based on the top 68 features in the importance ranking for recognition. This model was recorded as Model 7. For Model 7, the recognition accuracy of the training set was 93.55% and the recognition accuracy of the testing set was 90.38%. When the 1R method was used to select features, with increasing *K* value, the recognition accuracies of the training set and the testing set for the built KNN models fluctuated by 0.18%-3.72%. The optimal KNN model was built with a *K* value of 5 based on the top 71 features in the importance ranking for recognition, and this model was recorded as Model 8. For Model 8, the recognition accuracy of the training set was 92.36% and the recognition accuracy of the testing set was 88.93%. When the CFS method was used to select features, with increasing *K* value, the recognition accuracies of the training set and the testing set for the built KNN models fluctuated by 0.18%-2.09%. Based on the 21 selected features, the optimal KNN model was built with a *K* value of 5, and this model was recorded as Model 9. For Model 9, the recognition accuracy of the training set was 92.27% and the recognition accuracy of the testing set was 87.30%. With increasing *K* value, the recognition accuracies of the training set and the testing set for the built KNN models shown in Table 6 decreased in small-scale amplitude, indicating that the best *K* value in this study was 5. Considering the recognition accuracies of the training set and the testing set and the number of applied features for modeling, the optimality ranking of the three models shown in Table 6 was Model 7, Model 9, and Model 8.

**Table 6:** Recognition results for four alfalfa leaf diseases using KNN models based on selected features using the ReliefF method, the 1R method and the CFS method.

*K*	ReliefF method			1R method			CFS method		
	Recognition accuracy of training set (%)	Recognition accuracy of testing set (%)	Number of applied features	Recognition accuracy of training set (%)	Recognition accuracy of testing set (%)	Number of applied features	Recognition accuracy of training set (%)	Recognition accuracy of testing set (%)	Number of applied features
5	93.55	90.38	68	92.36	88.93	71	92.27	87.30	21
9	91.27	89.66	39	90.00	88.75	71	90.64	87.11	21
13	90.00	89.66	38	88.64	88.20	84	90.18	86.93	21

Note: Only the best KNN model for the image recognition of the four alfalfa leaf diseases is shown when the features were selected using the ReliefF method or 1R method.

#### Recognition Results of Disease Recognition Models Based on Semi-supervised Learning

**Fig 3:**
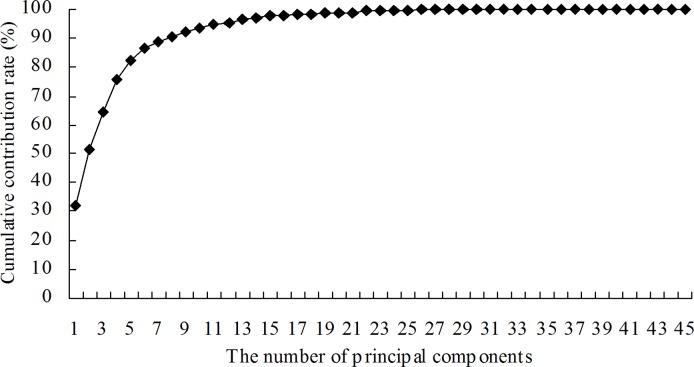
Changes in cumulative contribution rates with increasing number of principal components based on 45 features used for building Model 4.

Considering the recognition accuracies of the training set and the testing set and the number of applied features for modeling, Model 4 was regarded as the optimal model among the nine models described above. The recognition results of each type of alfalfa leaf disease using the optimal model are shown in Table 7. To eliminate the linear correlation between the features, the 45 features used for building Model 4 were transformed using PCA, and the changes in cumulative contribution rates with increasing number of principal components were achieved as shown in Fig 3. The results showed that the cumulative contribution rate of the first eight principal components reached 90.77% and that the cumulative contribution rate of the first 12 principal components reached 95.54%.

**Table 7:** Recognition results of each alfalfa leaf disease using the optimal model (Model 4).

Individual disease	Recognition accuracy of training set (%)	Recognition accuracy of testing set (%)
Alfalfa common leaf spot	89.19	75.00
Alfalfa rust	99.63	96.24
Alfalfa Leptosphaerulina leaf spot	97.30	96.76
Alfalfa Cercospora leaf spot	99.15	97.74

**Fig 4:**
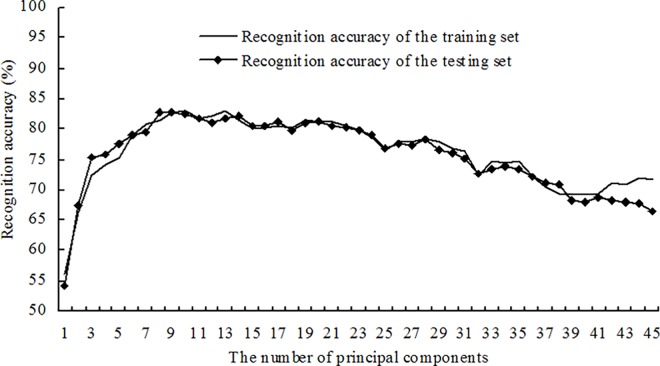
Recognition results for four alfalfa leaf diseases using semi-supervised models at a ratio of labeled to unlabeled samples of 2:1.

**Fig 5:**
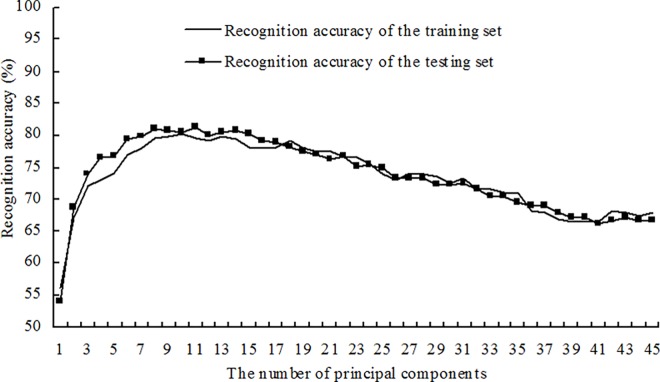
Recognition results for four alfalfa leaf diseases using semi-supervised models at a ratio of labeled to unlabeled samples of 1:1.

**Fig 6:**
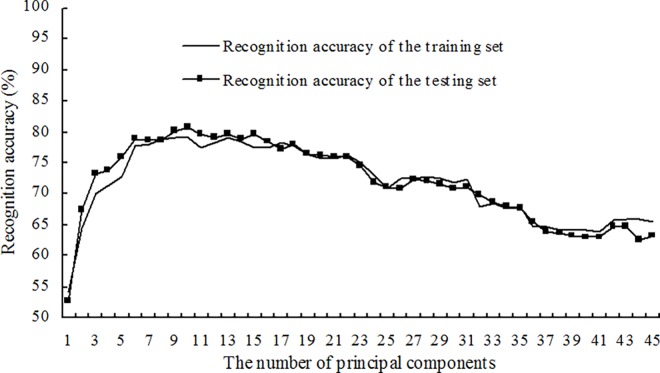
Recognition results for four alfalfa leaf diseases using semi-supervised models at a ratio of labeled to unlabeled samples of 1:2.

Based on the same training set and testing set as used for building the supervised models described above, the disease recognition semi-supervised models were built using a ratio of labeled to unlabeled samples in the training set equal to 2:1. The corresponding recognition accuracies of the training set and the testing set were obtained using the first *n* principal components as the inputs. The changes in recognition accuracies of the training set and testing set are shown in Fig 4 with an increased number of principal components. The results showed that for disease recognition semi-supervised models with a varying number of principal components, there were no obvious differences between the recognition accuracies of the training set and the recognition accuracies of the testing set. Moreover, both the recognition accuracy of the training set and the recognition accuracy of the testing set first increased and then decreased with increasing *n*. Similarly, the disease recognition semi-supervised models were built with ratios of labeled and unlabeled samples in the training set equal to 1:1 and 1:2. The first *n* principal components were used as the inputs, and the corresponding recognition accuracies of the training set and the testing set, as shown in Figs 5 and 6, were obtained. The results showed that the recognition accuracies of the training set and the testing set obtained using the semi-supervised models with the different ratios of labeled and unlabeled samples presented similar change tendencies.

The recognition results for the four alfalfa leaf diseases using optimal semi-supervised models with the different ratios of labeled and unlabeled samples are as shown in Table 8. The results showed that when the ratio of labeled to unlabeled samples was 2:1, the optimal semi-supervised model for disease recognition was built with the first nine principal components and was recorded as Model 10. For Model 10, the recognition accuracy of the training set was 82.82% and the recognition accuracy of the testing set was 82.76%. When the ratio of the labeled samples to the unlabeled samples was 1:1, the optimal semi-supervised model for disease recognition was built with the first ten principal components and was recorded as Model 11. For Model 11, the recognition accuracy of the training set was 80.36% and the recognition accuracy of the testing set was 80.58%. When the ratio of the labeled samples to the unlabeled samples was 1:2, the optimal semi-supervised model for disease recognition was built with the first ten principal components and was recorded as Model 12. For Model 12, the recognition accuracy of the training set was 79.18% and the recognition accuracy of the testing set was 80.58%. For Model 10, Model 11 and Model 12, the recognition accuracies of the training set and the testing set were all approximately 80%, indicating that the ratio of the labeled samples to the unlabeled samples in the training set had relatively small effects on the recognition results of the disease recognition semi-supervised models when the models were built with the three ratios.

**Table 8:** Recognition results of four alfalfa leaf diseases using optimal semi-supervised models with various ratios of labeled to unlabeled samples.

Model	The ratio of labeled samples to unlabeled samples	The number of Principal components	Cumulative contribution rate (%)	Recognition accuracy of training set (%)	Recognition accuracy of testing set (%)
Model 10	2:1	9	92.22	82.82	82.76
Model 11	1:1	10	93.45	80.36	80.58
Model 12	1:2	10	93.45	79.18	80.58

## Discussion

In this study, lesion image segmentation was conducted using the segmentation methods integrated with clustering algorithms and supervised classification algorithms. Compared to image segmentation methods using only clustering algorithms, there was no need to calculate and choose optimal clustering numbers for the clustering algorithms of the segmentation methods used in this study, which reduced computational costs. For the image segmentation methods using only the supervised classification algorithms, typical lesion pixels and typical health pixels are usually chosen from a large number of disease images to construct the training set. Based on this training set, a supervised classification model with general applicability is built for the lesion segmentation of all the disease images. There may be a certain degree of variation in the color of the lesion regions and the healthy regions of disease images due to the different causal agents and the different stages of disease development. This may result in difficulties in disease image recognition [45]. In the methods used in this study, a targeted training set containing typical lesion pixels and typical healthy pixels was constructed based on each sub-image, and the supervised classification model based on this training set was more suitable for lesion segmentation of this sub-image. However, these segmentation methods are only suitable for lesion segmentation of disease images in which the *H* component values of the lesion regions are less than the *H* component values of the healthy regions in HSV color space. Since there are many alfalfa leaf diseases with great differences in color between the lesions of different diseases, it is necessary to develop a lesion image segmentation method with a wider range of application in future studies.

In this study, a total of 129 texture, color and shape features were extracted for disease image recognition. Satisfactory recognition results were obtained using the disease recognition models built after feature selection, indicating that the features extracted from the lesion images could be effectively used to recognize and identify the four alfalfa leaf diseases. However, the 129 extracted features are commonly used in the field of image recognition and greatly differ from disease features used by plant disease experts during disease identification via naked-eye observation, resulting in a poor interpretation of the disease recognition models based on these extracted features. In future studies, attempts could be made to construct lesion image features suitable for certain plant diseases, according to the experience of plant diseases experts, in combination with image processing techniques.

In this study, the best recognition effects were observed in the SVM model based on the top 45 features in the importance ranking obtained using the ReliefF method. The recognition accuracy of the testing set was highest among all the models built in this study and was very close to the recognition accuracy of the training set, which indicated that this model not only could be used to obtain satisfactory recognition results but also had strong generalization ability. When the ReliefF method was used to conduct feature selection, the possible correlation between the features was not considered. However, the existence of the correlation could lead to the redundancy of features and increase the complexity of the disease recognition models. In further studies, the ReliefF method could be combined with the feature transformation methods such as PCA and independent component analysis to remove the correlation between the features, reduce the dimension of features and decrease the complexity of the disease recognition models.

Semi-supervised learning is a technique to conduct training and classification using a small number of labeled samples and a large number of unlabeled samples. In the field of image recognition, the cost of obtaining image samples is very low in some cases, but the cost of adding class labels to samples is very high. In this case, a semi-supervised learning method can be used to build an image recognition model to obtain satisfactory recognition results and reduce the cost of modeling. In research on plant disease image recognition, determining the true categories of diseases requires specialized agricultural technical personnel to conduct naked-eye observations, microscopic observation of morphological characteristics of causal agents, or pathogen detection using molecular biology techniques [7]. Thus, a large amount of manpower and material resources are usually required. Therefore, attempts were made to use semi-supervised learning methods to build image recognition models of alfalfa leaf diseases. The results showed that the recognition accuracies of the training set and the testing set were all approximately 80% for the optimal semi-supervised model when the proportion of the labeled samples in the training set was only 33.33% (i.e., the ratio of the labeled and unlabeled samples was 1:2). This indicated that it was feasible to build an image recognition model of alfalfa leaf diseases based on semi-supervised learning.

The image recognition of only four alfalfa leaf diseases was investigated in this study. Therefore, it is necessary to build a standard and comprehensive lesion image database to lay the foundation for the application of the automatic disease image recognition technology. In addition, the complex background of plant disease images poses great challenges for image segmentation and image recognition [45]. The images of alfalfa leaf diseases used in this study were taken on a white background in the laboratory. Further studies are needed to determine whether the image recognition methods used in this study are suitable for the automatic identification and diagnosis of alfalfa leaf diseases in nature.

Presently, the use of smart phones to take pictures and process data has become very powerful. Smart phone-based plant disease image recognition systems have been reported [46–49]. A mobile application could be developed using the optimal image recognition model of alfalfa leaf diseases built in this study to realize functions such as disease image acquisition, disease diagnosis and disease information sharing based on smart phone platforms. Such an application could facilitate disease management.

Generally, the diagnosis and identification of alfalfa leaf diseases are performed by agricultural experts or agricultural technicians mainly using the conventional diagnostic methods including naked-eye observations of disease symptoms and microscopic observations of morphological characteristics of causal agents. The accuracy and efficiency mainly depend on the experience of experts or technicians. It is subjective and time-consuming. When using PCR techniques to detect the infection of a specific alfalfa leaf disease, professional instruments, reagents and materials are required, and professional personnel are also required to perform operations [7, 50]. In addition, it will take some time to obtain detection results [7, 50]. With increasingly widespread applications of portable cameras or mobile phones with picture-taking features, it is easier to obtain camera equipment than PCR instruments. After image acquisition, it is only needed to input the image into a computer with a disease image recognition system, and then the results of disease identification can be achieved. This process does not require professional personnel or any chemical reagents. It is faster than PCR techniques to achieve identification results. Especially, when the computer image recognition system based on Internet or App (mobile application) based on smart phone is developed, it will be more convenient for image recognition of alfalfa leaf diseases. However, PCR techniques can play an important role in disease detection in the early stage of diseases, especially in detection of latently infected leaves without symptom appearance [50]. The identification and recognition of infected alfalfa leaves in the early stage of diseases using image recognition technology still need more investigations in future studies. Moreover, the method for identification of alfalfa leaf diseases in this study was developed based on the images of four types of alfalfa leaf diseases, it is necessary to conduct further research on evaluating this method with other leaf disorders to evaluate the risk of false positive.

## Conclusions

In this study, lesion image segmentation using the methods integrating with clustering algorithms and supervised classification algorithms, feature extraction of lesion images, feature normalization and feature selection were conducted. The disease recognition models were built by using pattern recognition methods. The satisfactory recognition results for four alfalfa leaf diseases were obtained. A feasible solution was provided for diagnosis and identification of alfalfa leaf diseases.

Among the twelve lesion segmentation methods integrating with clustering algorithms and supervised classification algorithms, the segmentation effects were best when the segmentation method integrating with the *K*_median clustering algorithm (from the clustering algorithms) and the linear discriminant analysis (from the supervised classification algorithms) was used based on an aggregated image dataset comprising 899 sub-images of four types of alfalfa leaf diseases. This segmentation method was thus used to carry out the segmentation of sub-images of four types of alfalfa leaf diseases for further feature extraction, feature normalization, feature selection and modeling.

A total of 129 texture, color and shape features were extracted from the 1,651 typical lesion images, each of which contained only one lesion. Attempts were made to conduct feature selection using three methods including the ReliefF method, the 1R method and the CFS method. The disease recognition models were built using three supervised learning methods, including random forest, SVM and KNN. The results demonstrated that the recognition effects were best in the SVM model based on the top 45 features in the importance ranking for recognition when the ReliefF method was used to conduct feature selection. For this model, the recognition accuracies of the training set and the testing set were 97.64% and 94.74%, respectively. In addition, after the 45 features used for building the model were transformed using PCA, the disease recognition semi-supervised models were constructed using a self-training algorithm based on Naive Bayes classifiers. For the optimal semi-supervised models built with ratios of labeled to unlabeled samples equal to 2:1, 1:1 and 1:2, the recognition accuracies of the training set and the testing set were all approximately 80%. The results indicated that it was feasible to identify and recognize four types of alfalfa leaf diseases using the solution provided in this study.

## Supporting Information

S1 Data Cumulative contribution rates with increase in number of principal components based on 45 features used for building Model 4. (XLSX)

S2 Data Recognition accuracies of the training set and the testing set using semi-supervised models with a different number of principal components (ratio of labeled to unlabeled samples was 2:1). (XLSX)

S3 Data Recognition accuracies of the training set and the testing set using semi-supervised models with a different number of principal components (ratio of labeled to unlabeled samples was 1:1). (XLSX)

S4 Data Recognition accuracies of the training set and the testing set using semi-supervised models with a different number of principal components (ratio of labeled to unlabeled samples was 1:2). (XLSX)
